# A multiplex biomarker assay improves the diagnostic performance of HE4 and CA125 in ovarian tumor patients

**DOI:** 10.1371/journal.pone.0240418

**Published:** 2020-10-19

**Authors:** Pia Leandersson, Anna Åkesson, Ingrid Hedenfalk, Susanne Malander, Christer Borgfeldt

**Affiliations:** 1 Department of Clinical Sciences, Obstetrics and Gynecology, Lund University, Reproductive Medicine Center, Skåne University Hospital Malmö, Malmo, Sweden; 2 Clinical Studies Sweden–Forum South, Skåne University Hospital Lund, Lund, Sweden; 3 Department of Clinical Sciences, Oncology and Pathology, Lund University, Lund, Sweden; 4 Department of Clinical Sciences, Oncology and Pathology, Lund University, Skåne University Hospital Lund, Lund, Sweden; 5 Department of Clinical Sciences, Obstetrics and Gynecology, Lund University, Skåne University Hospital Lund, Lund, Sweden; The Ohio State University, UNITED STATES

## Abstract

**Objective:**

Survival in epithelial ovarian cancer (EOC) remains poor. Most patients are diagnosed in late stages. Early diagnosis increases the chance of survival. We used the proximity extension assay from Olink Proteomics to search for new protein biomarkers with the potential to improve the diagnostic performance of CA125 and HE4 in patients with ovarian tumors.

**Material and methods:**

Plasma samples were obtained from 180 women with ovarian tumors; 30 cases of benign tumor, 28 cases with borderline tumors, 25 early EOC cases (FIGO stage I) and 97 advanced EOC cases (FIGO stages II-IV). Proteins were measured using the Olink® Oncology II and Inflammation panels. For statistical analyses, patients were categorized into benign tumors versus cancer and benign tumors versus borderline + cancer, respectively.

**Results:**

We analyzed 177 biomarkers. Thirty-four proteins had ROC AUC > 0.7 for discrimination between benign tumors and cancer. Fifteen proteins had ROC AUC > 0.7 for discrimination between benign tumors and borderline tumors + cancer. HE4 ranked highest for both comparisons. A reference model with HE4, CA125 and age (AUC 0.838 for benign tumors vs. cancer and AUC 0.770 for benign tumors vs. borderline tumors + cancer) was compared to the reference model with the addition of each of the remaining proteins with AUC > 0.7. ITGAV was the only individual biomarker found to improve diagnostic performance of the reference model, to AUC 0.874 for benign tumors vs. cancer and AUC 0.818 for benign tumors vs. borderline tumors + cancer (p < 0.05). Cross-validation and LASSO regression was combined to select multiple biomarker combinations. The best performing model for discrimination between benign tumors and borderline tumors + cancer was a 6-biomarker combination (HE4, CA125, ITGAV, CXCL1, CEACAM1, IL-10RB) and age (AUC 0.868, sensitivity 0.86 and specificity 0.82, p = 0.016 for comparison with the reference model).

**Conclusion:**

HE4 was the best performing individual biomarker for discrimination between benign ovarian tumors and EOC including borderline tumors. The addition of other carcinogenesis-related biomarkers in a multiplex biomarker panel can improve the diagnostic performance of the established biomarkers HE4 and CA125.

## Introduction

Around 700 Swedish women are diagnosed with ovarian cancer or borderline tumors every year. Symptoms are few and non-specific in the early stages, causing delays in diagnosis and treatment. While patients with borderline tumors have an excellent prognosis, with a five-year survival rate of 97%, the prognosis is poor in ovarian cancer patients. Half will die within five years of diagnosis [1,2].

Ovarian cancer is predominantly in the form of epithelial tumors (90%); the remaining 10% comprise germ cell and sex-cord stromal tumors. The main morphological subtypes in epithelial ovarian cancer (EOC) are high-grade serous (HGSC) (70%), endometrioid (EC) (10%), clear cell (CCC) (10%), mucinous (MC) (3%) and low grade serous cancer (LGSC) (<5%) [3]. These subtypes differ in origin and behavior, and respond very differently to oncological treatment [4,5]. Despite advances in surgical and oncological treatment, little improvement has been seen in long-term survival in EOC [6,7]. The majority of patients are diagnosed in late stages. In order to improve survival, the patients must be diagnosed earlier, when the disease is still curable. A screening method for ovarian cancer, for use in the general population, has been sought for decades. Two large-scale prospective population studies, the PLCO and UKCTOCS trials, were unable to show a significant decrease in ovarian cancer mortality from screening with the plasma protein biomarker CA125 and / or transvaginal ultrasound [8,9].

Apart from screening, a way to earlier ovarian cancer diagnosis is to improve the risk assessment when a patient presents with an adnexal mass. Patients with an estimated high risk of malignancy should be referred to the proper level of care without unnecessary delay. The multivariate Risk of Malignancy Index (RMI) algorithm (incorporating CA125, ultrasound score and menopause status) has been in clinical use since the 1990s [10]. The use of RMI requires ultrasound competence, which is not always available at the primary care level. In 2009, Moore et al. [11] introduced the Risk of Ovarian Malignancy Algorithm (ROMA) (CA125, HE4 and menopause status) dispensing with the need for ultrasound evaluation [11]. In their study comparing ROMA and RMI in 2010, Moore et al. [12] found better performance for ROMA compared to RMI, although these findings have been questioned by subsequent studies [12–15]. Both algorithms have reduced sensitivity and specificity in early stages of EOC when they would be of most diagnostic value [16]. Karlsen et al 2015 [17] introduced a modified version of the ROMA, the Copenhagen Index (CPH-I), substituting menopause status for age. The CPH-I, ROMA and RMI had comparable performance in a multicenter study [17]. However, ultrasound-based models have been found superior for the preoperative assessment of an adnexal mass, provided there is access to good quality ultrasonography [18]. Many research groups, including ours, have evaluated a range of other biomarkers and combinations of biomarkers for their potential use in ovarian cancer [19,20]. CA125 continues to stand out as the single-best biomarker [21,22] and considerable research has been focused on the search for additional biomarkers to improve the performance of CA125 alone [23]. Lately, researchers have turned to the rapidly evolving field of proteomics in the search for new candidate biomarkers, using new techniques for high throughput multiplex analysis in large-scale protein studies [24,25].

In this study we analyzed the Olink^®^ Oncology II and Inflammation panels (in total 177 unique protein biomarkers) in 180 women with benign tumor, borderline tumor, early (stage I) or late (stage II-IV) EOC, with the aim of searching for new candidate biomarkers with the potential to improve the performance of HE4 and CA125 for discrimination between benign disease and EOC. We tested the individual biomarkers in three-biomarker combinations with HE4, CA125 and age. Only the addition of ITGAV improved the reference model of HE4, CA125 and age to a significant level. In order to test whether a multiplex biomarker model could further improve performance of the reference model, we combined cross-validation with LASSO regression. A 6-biomarker model (HE4, CA125, CXCL1, ITGAV, CEACAM1, IL-10RB and age) was found to be the best model for discrimination between benign tumors and EOC including borderline tumors.

## Material and methods

A single cohort-design was used for biomarker discovery, with the aim of validation in a larger subsequent cohort of patients in case of positive findings.

Peripheral blood samples were obtained preoperatively from 180 women with an adnexal mass admitted for surgery at the Department of Obstetrics and Gynecology, Skåne University Hospital Lund, Sweden 2005 to 2012. Blood was collected in citrate tubes, centrifuged, and then the plasma was stored at −20°C until it was analyzed. All diagnoses were verified by histopathologic examination. The histological type and stage of the disease according to the International Federation of Gynecology and Obstetrics (FIGO) were available in all malignant cases. The patient cohort included 30 cases of benign adnexal mass, 28 cases with borderline tumors, 25 early EOC cases (FIGO stage I) and 97 advanced EOC cases (FIGO stage II-IV) (Table 1). The frozen plasma samples were shipped to Olink Proteomics AB, Uppsala, Sweden, for analyses.

**Table 1:** Patient cohort.

Tumor category	Benign	Borderline	EOC stage I	EOC stage II-IV	Total
**N** (%)	30 (16.7%)	28 (15.6%)	25 (13.9%)	97 (53.9%)	180 (100%)
**Mean age** (range)	54^a^ (24–87)	51 (26–84)	61 (27–87)	66 (35–88)	61 (24–88)
**Histology**					
Serous	13	13	8	88	122
Mucinous	6	12	5	3	26
Endometrioid	7	3	10	5	25
Clear cell			2	1	3
Teratoma	4				4

^a^ Information on age /date of surgery not available in one patient with a benign tumor.

### Proximity extension assay

Proteins were measured using the Olink® Oncology II and Inflammation panels (Olink Proteomics AB, Uppsala, Sweden) according to the manufacturer's instructions. The biomarkers included in each panel are listed in the supporting information, in the S1 and S2 Files. The Proximity Extension Assay (PEA) technology used for the Olink protocol has been well described [26] and enables 92 analytes to be analyzed simultaneously, using 1 μL of each sample. Pairs of oligonucleotide-labeled antibody probes bind to their targeted protein, and if the two probes are brought into close proximity the oligonucleotides will hybridize in a pair-wise manner. The addition of a DNA polymerase leads to a proximity-dependent DNA polymerization event, generating a unique PCR target sequence. The resulting DNA sequence is subsequently detected and quantified using a microfluidic real-time PCR instrument (Biomark HD, Fluidigm). The final assay read-out is presented in Normalized Protein eXpression (NPX) values, which is an arbitrary unit on a log2-scale where a high value corresponds to a higher protein expression. The NPX values are relative and not comparable between different proteins.

Analyses were performed by biomedical technicians at Olink Proteomics AB in Uppsala, Sweden. Disease status of the patients was unknown to the technicians performing the analyses. Samples were randomized across the plates and run in duplicates. Data was quality controlled and normalized using an internal extension control and an inter-plate control, to adjust for intra- and inter-run variation. All assay validation data (detection limits, intra- and inter-assay precision data, etc.) are available on the manufacturer's website (www.olink.com).

### Statistical analyses

Hierarchical clustering analysis and principal component analysis were performed to search for clusters of proteins associated with the different tumor categories. Patients were subsequently categorized into benign tumors versus cancer, or benign tumors versus borderline tumors and cancer, and differences in protein expression between groups were analyzed with a Student’s t-test with a p-value < 0.001 indicating a statistically significant difference; p-values were adjusted for multiple comparisons using the False Discovery Rate (FDR). Each biomarker was used as a continuous variable in univariate logistic regression models, with the binary outcome benign tumors versus cancer or benign tumors versus borderline tumors and cancer. Receiver Operator Curves (ROC) were constructed and the Area under the Curve (AUC) was calculated with 95% confidence intervals using the non-parametric bootstrap procedure. In order to evaluate the biomarkers’ potential to improve the performance of the ROMA and CPH-I algorithms, a multivariate logistic regression model including the biomarkers HE4 and CA125 and age was constructed to serve as a reference model. Each of the biomarkers with AUC > 0.7 was added in turn to the reference model. The classification accuracy of each model was evaluated with the AUC, and the AUC for each model was compared to the AUC of the reference model using DeLong’s method. A p-value < 0.05 for differences in AUC was considered statistically significant. For each model, the sensitivity corresponding to a specificity of 0.95, and specificity corresponding to sensitivity of 0.95 was calculated.

We wished to test whether a multiplex biomarker model could further improve diagnostic performance of the reference model. In order to select which combination of biomarkers to include in a final logistic regression model in addition to HE4, and CA-125 and age, a combination of cross-validation and LASSO-regression was employed. To start with the data was randomly split suing a 50/50 split into a training and test set. In the training set the shrinkage parameter (λ) was estimated using k-fold cross-validation. The estimated shrinkage parameter λ_CV_ was then used in the test set in order to perform variable selection. The selected variables and the absolute value of the coefficients were saved. This process was then repeated 10 times. Next, the variables were ordered by the number of times they were selected and the sum of its estimated coefficients. The lowest ranked variable was removed and the entire process was repeated until a final model was selected. The final models were estimated with logistic regression. Receiver Operator Curves (ROC) were constructed and Area under Curve (AUC) calculated with 95% confidence intervals using the non-parametric bootstrap procedure. The AUC for each model was compared to the AUC of the reference model using DeLong’s method. A p-value < 0.05 for differences in AUC was considered statistically significant.

All statistical analyses were carried out using R v 4.0.0 (R Core Team (2018). R: A language and environment for statistical computing. R Foundation for Statistical Computing, Vienna, Austria. URL http://www.R-project.org/).

### Ethics statement

Written informed consent was obtained from all study participants. Ethical approval was granted by the Ethical Review Board at the Faculty of Medicine, Lund University, Sweden. Dnr 495 2016 (amendment to Dnr 558–2004 and 94–2006).

## Results

Out of the 180 patient samples, eight samples did not pass internal quality control in the PEA analyses (www.olink.com) and were excluded from statistical analyses. These samples comprised two borderline tumors and six advanced stage EOC cases. The analyses below include 172 patients.

Non-hierarchical clustering analysis was performed for the whole patient cohort and for serous tumors alone. Heat maps indicating protein expression levels for each patient are shown in the S1 Fig. No clustering could be observed visually. Principal component analysis did not segregate the patients into groups according to protein expression levels (S2 Fig).

### Benign tumors vs. cancer

**Fig 1:**
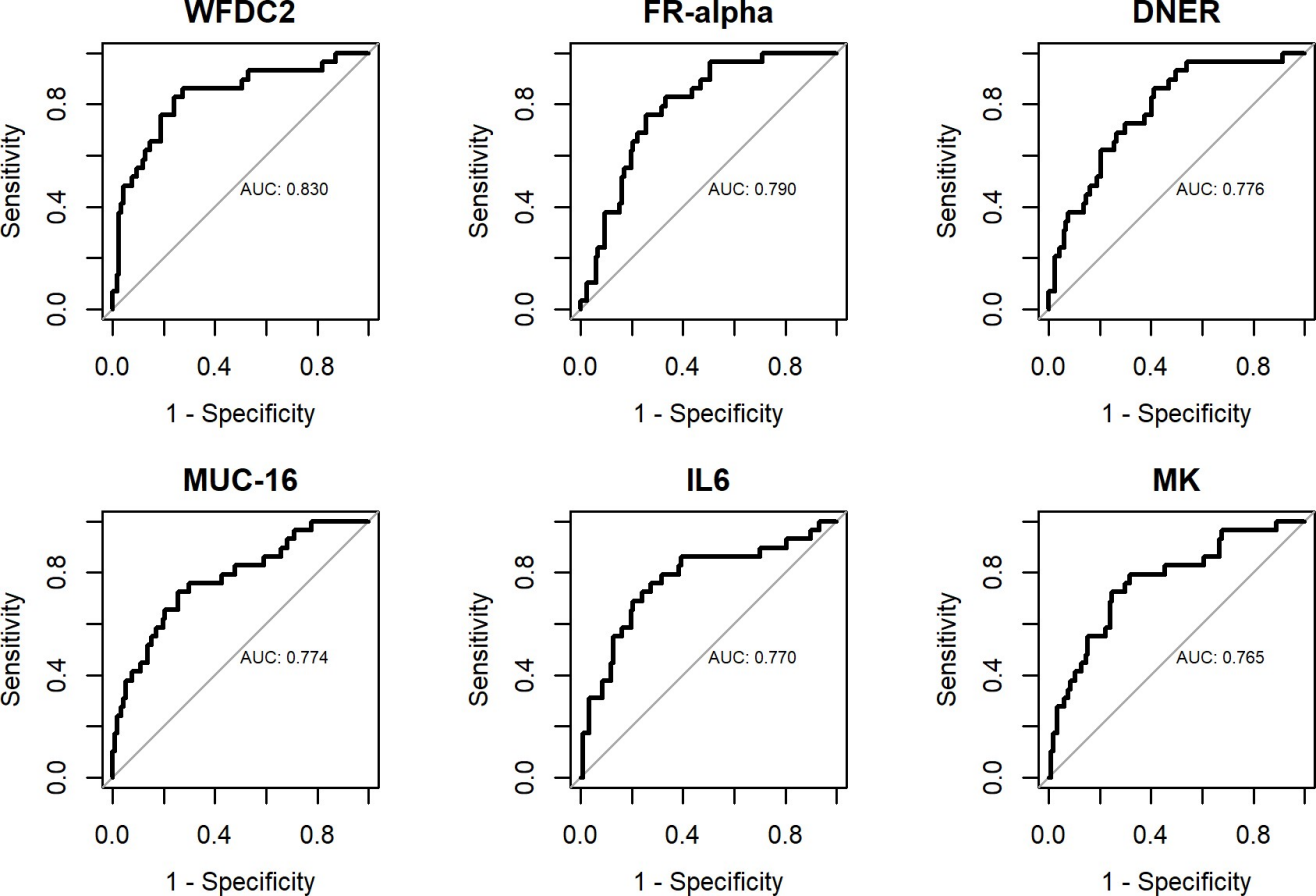
ROC curves for individual biomarkers. Benign tumors vs. cancer.

Out of the 177 biomarkers analyzed, a statistically significant difference in NPX levels between benign tumors and cancer was found for eight proteins (using a conservative cut-off p < 0.001, p-values adjusted with False Discovery Rate (FDR)). HE4 (WFDC2) and CA125 (MUC16) were highest ranked. Most of the proteins were up-regulated in cancer patients although lower NPX levels were seen for two proteins, ITGAV and DNER, in cancer patients (Table 2). Box plots for the six proteins with the lowest p-values are shown in S3 Fig. Table 3 shows the AUC values for discriminating benign tumors from cancer for the individual proteins. 34 proteins had AUC > 0.7. HE4 (WFDC2) ranked highest with AUC 0.830 (95% CI 0.739–0.921). ROC curves for the six proteins with the highest AUC values are depicted in Fig 1. Table 4 shows the AUC values, sensitivities (95% specificity) and specificities (95% sensitivity) for the reference model with HE4, CA125 and age (AUC 0.838 (0.752–0.924) and for the reference model with the addition of each one of the remaining 32 proteins with AUC > 0.7. ITGAV was the only biomarker to significantly improve the diagnostic performance of the reference model, to AUC 0.874 (0.799–0.949) (p = 0.045). Sensitivities and specificities are low with wide confidence intervals, calling for caution when interpreting results.

**Table 2:** Normalized protein expression (NPX) values. Benign tumors vs. cancer, p < 0.05.

	Benign	Cancer		
Protein	Mean (sd)	Mean (sd)	Adj. p-val^a^	Rank
WFDC2	7.37 (0.72)	8.33 (0.64)	<0.001	1
MUC-16	4.73 (1.93)	6.55 (1.40)	<0.001	2
MK	7.15 (0.86)	8.04 (0.84)	<0.001	3
FR-alpha	7.00 (0.75)	8.31 (1.36)	<0.001	4
ITGAV	3.38 (0.27)	3.05 (0.34)	<0.001	5
DNER	8.03 (0.37)	7.57 (0.48)	<0.001	6
IL6	4.07 (1.31)	5.35 (1.40)	<0.001	7
CDCP1	3.55 (0.72)	4.26 (0.81)	<0.001	8
VEGFR-2	7.13 (0.28)	6.82 (0.35)	0.001	9
IL10	3.02 (0.61)	3.76 (0.88)	0.001	10
CXCL13	8.38 (0.80)	9.10 (0.84)	0.001	11
WISP-1	4.48 (0.60)	5.16 (0.81)	0.001	12
TNFRSF6B	4.32 (0.71)	5.03 (0.85)	0.001	13
CTSV	3.76 (0.71)	3.14 (0.71)	0.001	14
CCL23	10.04 (0.51)	10.48 (0.52)	0.001	15
CXCL1	7.66 (1.34)	8.67 (1.22)	0.002	16
SCF	9.69 (0.52)	9.09 (0.79)	0.002	17
TNFRSF4	3.32 (0.60)	3.85 (0.69)	0.002	18
CXCL11	8.10 (1.27)	9.29 (1.58)	0.002	19
VEGFA	9.88 (0.56)	10.45 (0.79)	0.003	20
CD40	9.93 (0.68)	10.55 (0.88)	0.005	21
IL-10RB	6.74 (0.34)	7.04 (0.42)	0.005	22
CSF-1	8.17 (0.30)	8.38 (0.28)	0.005	23
TNFSF14	4.53 (0.77)	5.26 (1.08)	0.005	24
CCL3	4.91 (0.70)	5.54 (0.92)	0.005	25
SCAMP3	3.03 (1.82)	4.61 (2.33)	0.006	26
CXCL6	7.53 (1.29)	8.50 (1.42)	0.006	27
ABL1	2.79 (1.63)	4.32 (2.37)	0.008	28
TXLNA	4.01 (1.92)	5.58 (2.42)	0.008	29
IL-17C	1.48 (0.63)	2.30 (1.32)	0.008	30
PVRL4	6.10 (0.65)	6.75 (1.03)	0.008	31
IL8	6.26 (1.26)	7.04 (1.13)	0.008	32
IL-18R1	7.05 (0.47)	7.41 (0.55)	0.008	33
RSPO3	3.81 (0.61)	4.37 (0.89)	0.008	34
TFPI-2	7.78 (0.63)	8.31 (0.82)	0.008	35
MCP-1	10.14 (0.55)	10.58 (0.69)	0.008	36
CXCL9	7.70 (1.24)	8.46 (1.13)	0.008	37
ICOSLG	3.90 (0.29)	3.65 (0.39)	0.008	38
PD-L1	3.79 (0.45)	4.18 (0.61)	0.008	39
TNFRSF9	6.20 (0.63)	6.66 (0.73)	0.008	40
IL-15RA	0.35 (0.18)	0.50 (0.24)	0.008	41
DKN1A	2.36 (2.33)	4.19 (2.95)	0.009	42
LYN	1.17 (1.11)	1.94 (1.27)	0.011	43
LYPD3	4.07 (0.48)	3.70 (0.61)	0.011	44
FASLG	8.90 (0.57)	8.54 (0.57)	0.012	45
ADAM-TS 15	4.33 (0.58)	4.76 (0.71)	0.012	46
OPG	10.66 (0.30)	10.94 (0.48)	0.012	47
ESM-1	8.92 (0.48)	9.25 (0.56)	0.014	48
MMP-1	12.32 (1.37)	13.36 (1.84)	0.018	49
EGF	6.25 (2.23)	7.74 (2.62)	0.018	50
SYND1	6.46 (0.67)	6.94 (0.85)	0.018	51
MSLN	2.18 (0.88)	2.89 (1.33)	0.021	52
IL7	3.59 (1.21)	4.35 (1.39)	0.023	53
LAP TGF-beta-1	7.53 (0.67)	8.00 (0.88)	0.023	54
TNFSF13	8.39 (0.39)	8.65 (0.47)	0.025	55
VIM	3.47 (1.32)	4.07 (1.01)	0.025	56
IL-12B	4.38 (0.70)	4.80 (0.77)	0.027	57
SEZ6L	5.33 (0.40)	5.06 (0.51)	0.027	58
hK11	5.79 (0.77)	6.35 (1.07)	0.027	59
SIRT2	3.38 (1.99)	4.66 (2.46)	0.028	60
EPHA2	1.46 (0.46)	1.79 (0.64)	0.028	61
4E-BP1	8.82 (1.31)	9.52 (1.29)	0.028	62
TNFRSF19	4.06 (0.49)	4.46 (0.77)	0.028	63
FURIN	4.02 (0.46)	4.29 (0.50)	0.029	64
CCL4	6.44 (0.60)	6.91 (0.93)	0.029	65
FADD	0.75 (1.22)	1.52 (1.51)	0.029	66
AXIN1	3.55 (2.20)	5.03 (2.93)	0.029	67
MCP-4	3.74 (1.10)	4.31 (1.08)	0.029	68
CEACAM1	7.56 (0.17)	7.43 (0.23)	0.029	69
CD27	7.85 (0.43)	8.14 (0.58)	0.029	70
HGF	8.39 (0.54)	8.78 (0.79)	0.031	71
FGF-21	5.82 (1.52)	6.69 (1.72)	0.034	72
STAMPB	4.90 (1.67)	5.96 (2.15)	0.034	73
MetAP 2	4.18 (1.06)	4.79 (1.25)	0.035	74
CASP-8	1.19 (0.76)	1.69 (1.04)	0.038	75
TNFB	3.92 (0.39)	3.60 (0.72)	0.043	76
CCL20	5.59 (1.22)	6.19 (1.28)	0.049	77
ST1A1	1.16 (1.29)	1.94 (1.69)	0.049	78

^a^ p-values adjusted with FDR.

**Table 3:** AUC for individual biomarkers. Benign tumors vs. cancer, AUC > 0.7.

Protein	AUC (95% CI)	Rank
WFDC2	0.830 (0.739–0.921)	1
FR-alpha	0.790 (0.709–0.870)	2
DNER	0.776 (0.687–0.866)	3
MUC-16	0.774 (0.676–0.872)	4
IL6	0.770 (0.664–0.876)	5
MK	0.765 (0.666–0.863)	6
VEGFR-2	0.764 (0.661–0.867)	7
IL10	0.763 (0.670–0.855)	8
ITGAV	0.761 (0.673–0.849)	9
WISP-1	0.757 (0.665–0.849)	10
TNFRSF6B	0.747 (0.648–0.845)	11
SCF	0.747 (0.646–0.847)	12
CXCL13	0.744 (0.640–0.849)	13
CDCP1	0.743 (0.647–0.839)	14
RSPO3	0.728 (0.622–0.833)	15
ABL1	0.726 (0.625–0.827)	16
CCL23	0.726 (0.616–0.835)	17
CXCL6	0.726 (0.611–0.840)	18
MMP-1	0.723 (0.624–0.822)	19
CXCL11	0.722 (0.619–0.826)	20
CTSV	0.722 (0.620–0.825)	21
CD40	0.719 (0.617–0.821)	22
VEGFA	0.718 (0.617–0.819)	23
CXCL9	0.716 (0.603–0.829)	24
CXCL1	0.713 (0.602–0.825)	25
TFPI-2	0.712 (0.607–0.817)	26
CSF-1	0.710 (0.598–0.823)	27
CCL3	0.710 (0.603–0.817)	28
TNFB	0.710 (0.610–0.810)	29
TNFRSF4	0.708 (0.609–0.807)	30
SCAMP3	0.706 (0.606–0.807)	31
TNFSF14	0.705 (0.606–0.804)	32
PVRL4	0.704 (0.609–0.799)	33
IL-10RB	0.703 (0.602–0.803)	34

**Table 4:** Three-biomarker models. Benign tumors vs. cancer.

Reference model	AUC (95%)	Rank	p-value^a^	Sensitivity at 95% specificity	Specificity at 95% sensitivity
HE4+CA125+age	0.838 (0.752–0.924)	-	-	0.483 (0.207–0.759)	0.350 (0.111–0.709)
Additional marker					
CXCL1	0.874 (0.803–0.945)	1	0.068	0.448 (0.207–0.655)	0.581 (0.137–0.803)
ITGAV	0.874 (0.799–0.949)	2	0.045	0.621 (0.207–0.862)	0.530 (0.188–0.709)
CXCL6	0.872 (0.803–0.942)	3	0.150	0.448 (0.138–0.724)	0.444 (0.325–0.821)
SCAMP3	0.869 (0.797–0.942)	4	0.094	0.379 (0.172–0.724)	0.462 (0.239–0.787)
ABL1	0.868 (0.794–0.942)	5	0.111	0.414 (0.172–0.724)	0.385 (0.265–0.786)
VEGFR-2	0.863 (0.777–0.948)	6	0.304	0.586 (0.172–0.793)	0.291 (0.145–0.701)
CD40	0.859 (0.784–0.935)	7	0.154	0.414 (0.138–0.724)	0.444 (0.231–0.769)
CXCL11	0.859 (0.780–0.937)	8	0.197	0.414 (0.207–0.690)	0.316 (0.197–0.744)
DNER	0.858 (0.775–0.941)	9	0.157	0.586 (0.310–0.793)	0.564 (0.026–0.684)
SCF	0.858 (0.774–0.942)	10	0.246	0.517 (0.276–0.724)	0.342 (0.060–0.744)
TNFSF14	0.851 (0.770–0.931)	11	0.304	0.379 (0.172–0.724)	0.299 (0.188–0.744)
CTSV	0.849 (0.764–0.934)	12	0.526	0.552 (0.241–0.759)	0.333 (0.137–0.752)
CXCL13	0.848 (0.771–0.925)	13	0.363	0.414 (0.103–0.690)	0.496 (0.197–0.709)
FR-alpha	0.846 (0.769–0.924)	14	0.471	0.483 (0.138–0.759)	0.444 (0.274–0.684)
MMP-1	0.846 (0.762–0.930)	15	0.318	0.517 (0.138–0.725)	0.359 (0.085–0.744)
IL10	0.842 (0.758–0.926)	16	0.543	0.448 (0.069–0.725)	0.350 (0.103–0.735)
TNFB	0.842 (0.754–0.930)	17	0.715	0.483 (0.241–0.759)	0.316 (0.085–0.701)
CSF-1	0.841 (0.756–0.925)	18	0.409	0.483 (0.207–0.724)	0.393 (0.137–0.692)
CXCL9	0.840 (0.756–0.925)	19	0.759	0.483 (0.172–0.759)	0.316 (0.197–0.692)
IL-10RB	0.840 (0.760–0.920)	20	0.779	0.448 (0.103–0.690)	0.504 (0.154–0.684)
PVRL4	0.839 (0.754–0.925)	21	0.846	0.517 (0.207–0.759)	0.402 (0.171–0.692)
TNFRSF6B	0.839 (0.753–0.925)	22	0.143	0.483 (0.207–0.759)	0.350 (0.111–0.709)
WISP-1	0.839 (0.753–0.925)	23	0.775	0.448 (0.207–0.724)	0.359 (0.085–0.701)
CCL3	0.838 (0.752–0.925)	24	0.565	0.517 (0.207–0.759)	0.355 (0.103–0.684)
CDCP1	0.838 (0.753–0.924)	25	0.893	0.483 (0.172–0.759)	0.368 (0.103–0.701)
TNFRSF4	0.838 (0.755–0.921)	26	0.962	0.483 (0.207–0.724)	0.410 (0.137–0.692)
CCL23	0.838 (0.750–0.925)	27	1.000	0.448 (0.172–0.724)	0.376 (0.077–0.701)
MK	0.837 (0.751–0.924)	28	0.930	0.448 (0.138–0.724)	0.308 (0.120–0.692)
RSPO3	0.837 (0.752–0.923)	29	0.900	0.448 (0.172–0.724)	0.333 (0.094–0.701)
VEGFA	0.837 (0.749–0.925)	30	0.836	0.414 (0.138–0.724)	0.299 (0.068–0.718)
TFPI-2	0.836 (0.749–0.924)	31	0.737	0.483 (0.207–0.724)	0.342 (0.060–0.692)
IL6	0.834 (0.743–0.926)	32	0.647	0.483 (0.172–0.759)	0.282 (0.026–0.684)

^a^ Comparing the reference model and the reference model with an added biomarker.

**Fig 2:**
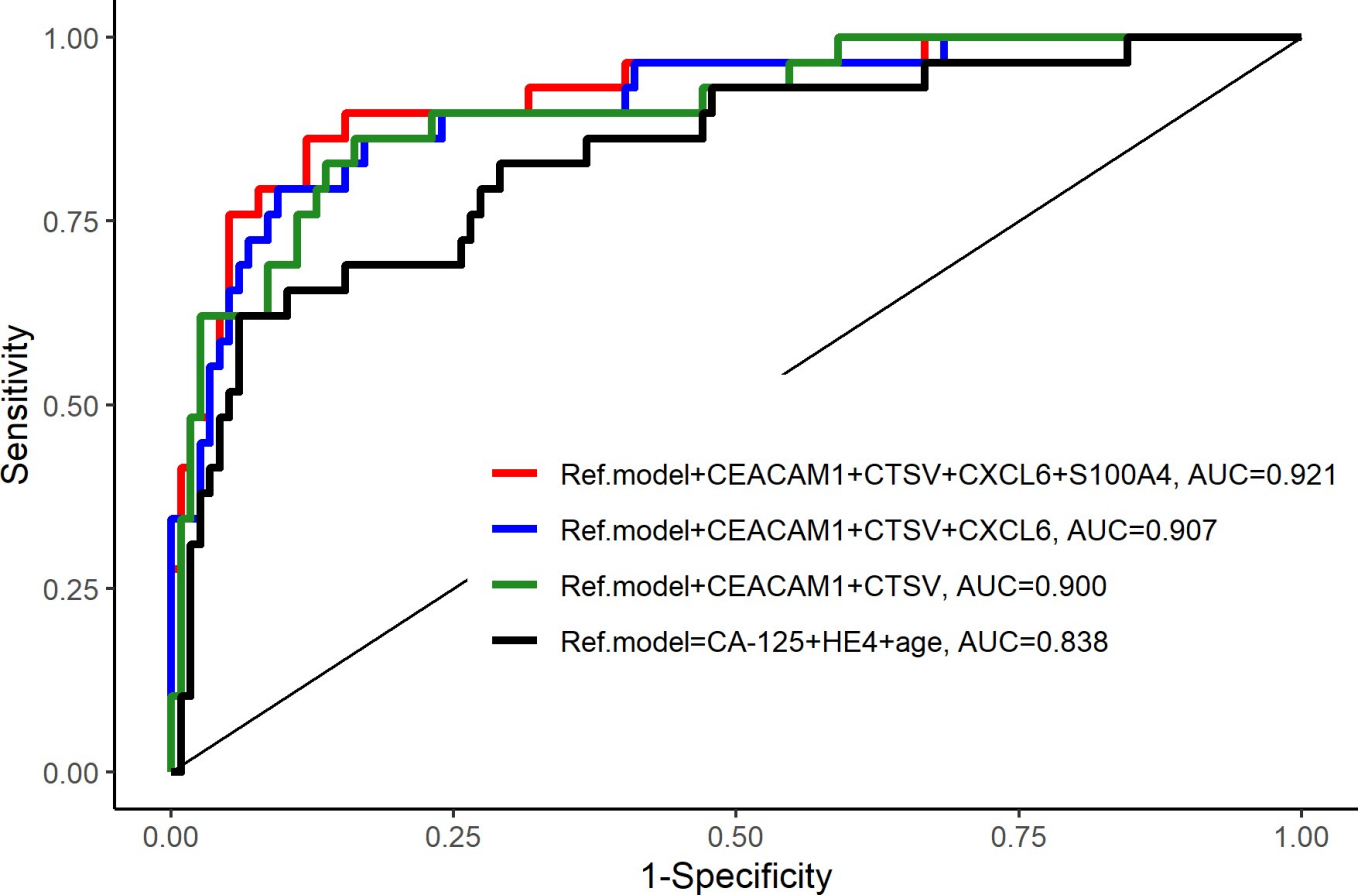
ROC curves for biomarker models. Benign tumors vs. cancer.

Cross-validation and LASSO regression were used to select multi-biomarker combinations for logistic regression models. A six-biomarker model including HE4, CA125, CEACAM1, CTSV, CXCL6, S100A4 and age was found to be the best model for discriminating between benign tumors and EOC with AUC 0.921 (0.863–0.979), sensitivity 0.897 / specificity 0.889 at best point cut-off (p = 0.025) (Table 5 and Fig 2).

**Table 5:** Biomarker models, LASSO regression. Benign tumors vs. cancer.

Reference model	AUC (95%)	p-value^a^	Sensitivity at 95% specificity	Specificity at 95% sensitivity	Specificity at best point	Sensitivity at best point se
HE4 + CA125+ age	0.838 (0.752–0.924)	-	0.483 (0.207–0.759)	0.350 (0.111–0.701)	0.987 (0.590–0.974)	0.759 (0.552–0.966)
Additional marker combinations						
CEACAM1+CTSV+CXCL6+S100A4	0.921 (0.863–0.979)	0.025	0.655 (0.379–0.897)	0.615 (0.282–0.906)	0.889 (0.803–0.974)	0.897 (0.724–0.966)
CEACAM1+CTSV+CXCL6	0.907 (0.845–0.970)	0.039	0.621 (0.276–0.862)	0.581 (0.256–0.863)	0.889 (0.735–0.957)	0.862 (0.690–0.966)
CEACAM1+CXCL6	0.900 (0.836–0.965)	0.061	0.621 (0.379–0.793)	0.479 (0.350–0.793)	0.855 (0.752–0.974)	0.862 (0.687–0.966)

^a^ Comparing the reference model and the reference model with additional marker combinations.

### Benign tumors vs. borderline + cancer

**Fig 3:**
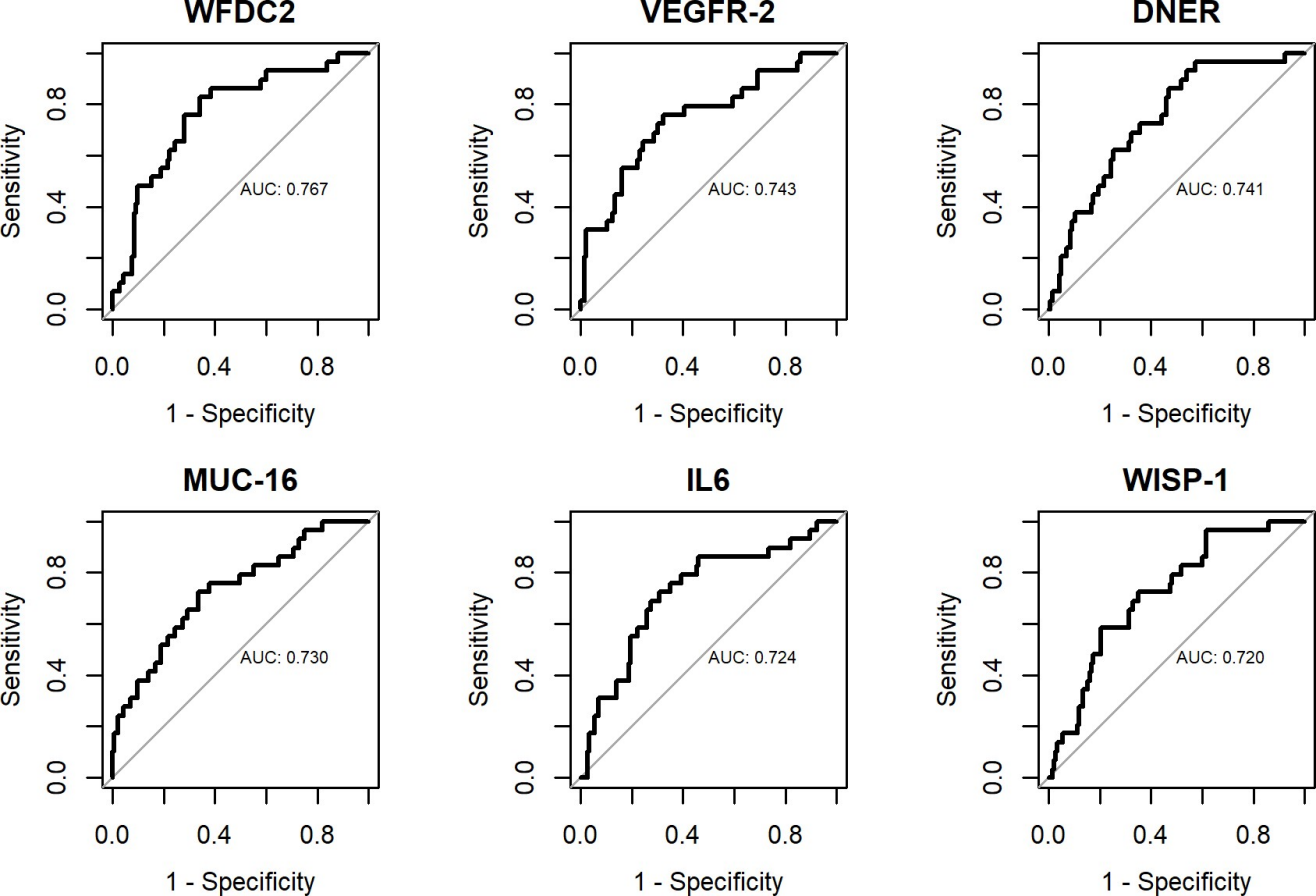
ROC curves for individual biomarkers. Benign tumors vs. borderline + cancer.

A statistically significant difference in NPX levels between benign tumors and borderline tumors + cancer was found for only two proteins, HE4 (WFDC2) and CA125 (MUC16) (conservative cut-off p < 0.001, p-values adjusted with False Discovery Rate (FDR)) (Table 6). Box plots for the six proteins with the lowest p-values are shown in S4 Fig. Table 7 shows the AUC values for discriminating benign tumors from borderline tumors + cancer for the individual proteins. Fifteen proteins had AUC > 0.7. HE4 (WFDC2) ranked highest with AUC 0.767 (0.672–0.861). ROC curves for the six proteins with the highest AUC values are depicted in Fig 3. Table 8 shows the AUC values, sensitivities and specificities for the reference model with HE4, CA125 and age (AUC 0.770 (0.674–0.865)) and for the reference model with the addition of each one of the remaining 13 proteins with AUC > 0.7. Again, only the addition of ITGAV would significantly increase the diagnostic performance of the reference model, to AUC 0.818 (0.737–0.900) (p<0.05). For both models the sensitivities and specificities were low and confidence intervals wide, indicating statistical uncertainty.

**Table 6:** Normalized protein expression (NPX) values. Benign tumors vs. borderline + cancer, p < 0.05.

	Benign	Borderline + Cancer		
Protein	Mean (sd)	Mean (sd)	Adj. p-val^a^	Rank
WFDC2	7.35 (0.71)	8.15 (0.75)	<0.001	1
MUC-16	4.67 (1.93)	6.26 (1.54)	<0.001	2
ITGAV	3.37 (0.27)	3.08 (0.35)	0.002	3
MK	7.11 (0.88)	7.86 (0.92)	0.002	4
VEGFR-2	7.13 (0.28)	6.85 (0.35)	0.002	5
FR-alpha	6.99 (0.75)	8.05 (1.41)	0.002	6
DNER	8.01 (0.37)	7.63 (0.49)	0.002	7
CTSV	3.75 (0.70)	3.19 (0.70)	0.002	8
CXCL13	8.37 (0.78)	9.02 (0.82)	0.002	9
WISP-1	4.45 (0.61)	5.06 (0.81)	0.003	10
IL6	4.03 (1.31)	5.15 (1.51)	0.003	11
CXCL1	7.56 (1.42)	8.55 (1.30)	0.004	12
CCL23	10.03 (0.51)	10.42 (0.54)	0.004	13
CXCL11	8.06 (1.27)	9.12 (1.55)	0.007	14
TNFRSF6B	4.33 (0.70)	4.90 (0.86)	0.008	15
CDCP1	3.52 (0.72)	4.09 (0.88)	0.010	16
IL10	3.03 (0.60)	3.61 (0.92)	0.011	17
IL-10RB	6.73 (0.34)	7.00 (0.42)	0.012	18
SCF	9.67 (0.51)	9.19 (0.78)	0.012	19
VEGFA	9.85 (0.58)	10.34 (0.80)	0.013	20
CD40	9.91 (0.69)	10.47 (0.90)	0.014	21
CXCL6	7.45 (1.32)	8.37 (1.46)	0.014	22
ICOSLG	3.90 (0.29)	3.67 (0.39)	0.015	23
IL-18R1	7.07 (0.47)	7.38 (0.51)	0.015	24
IL-17C	1.44 (0.66)	2.17 (1.26)	0.015	25
CCL3	4.88 (0.70)	5.45 (0.96)	0.015	26
PD-L1	3.78 (0.45)	4.13 (0.60)	0.015	27
MMP-1	12.26 (1.39)	13.30 (1.76)	0.015	28
TNFRSF4	3.34 (0.60)	3.75 (0.69)	0.015	29
ABL1	2.76 (1.62)	4.16 (2.42)	0.015	30
LYPD3	4.08 (0.47)	3.73 (0.58)	0.015	31
SCAMP3	2.98 (1.80)	4.39 (2.40)	0.015	32
IL-15RA	0.34 (0.18)	0.48 (0.24)	0.015	33
TNFSF14	4.52 (0.76)	5.15 (1.13)	0.018	34
TXLNA	3.96 (1.91)	5.36 (2.46)	0.018	35
CSF-1	8.16 (0.30)	8.33 (0.29)	0.018	36
IL8	6.21 (1.28)	6.90 (1.17)	0.019	37
DKN1A	2.30 (2.31)	3.96 (2.97)	0.019	38
RSPO3	3.80 (0.61)	4.28 (0.86)	0.019	39
LYN	1.14 (1.10)	1.85 (1.28)	0.024	40
MCP-1	10.12 (0.56)	10.49 (0.69)	0.024	41
LAP TGF-beta-1	7.49 (0.69)	7.96 (0.87)	0.025	42
PVRL4	6.09 (0.63)	6.63 (1.01)	0.025	43
TFPI-2	7.78 (0.61)	8.22 (0.81)	0.026	44
IL7	3.53 (1.23)	4.27 (1.38)	0.026	45
MCP-4	3.69 (1.12)	4.29 (1.11)	0.026	46
EGF	6.18 (2.23)	7.57 (2.62)	0.026	47
CXCL9	7.68 (1.23)	8.31 (1.17)	0.029	48
CEACAM1	7.55 (0.17)	7.43 (0.23)	0.030	49
ADAM-TS 15	4.32 (0.58)	4.68 (0.69)	0.033	50
FASLG	8.91 (0.57)	8.61 (0.58)	0.033	51
OPG	10.65 (0.31)	10.89 (0.49)	0.036	52
CCL4	6.42 (0.61)	6.87 (0.95)	0.041	53
CXCL5	9.69 (2.15)	10.70 (2.02)	0.046	54
IL-12B	4.40 (0.69)	4.76 (0.74)	0.046	55
ESM-1	8.92 (0.47)	9.19 (0.57)	0.047	56
SIRT2	3.30 (2.00)	4.50 (2.53)	0.048	57

^a^ p-values adjusted with FDR.

**Table 7:** AUC for individual biomarkers. Benign tumors vs. borderline + cancer, AUC > 0.7.

Protein	AUC (95% CI)	Rank
WFDC2	0.767 (0.672–0.861)	1
VEGFR-2	0.743 (0.639–0.848)	2
DNER	0.741 (0.650–0.833)	3
ITGAV	0.731 (0.639–0.822)	4
MUC-16	0.730 (0.628–0.833)	5
IL6	0.724 (0.619–0.829)	6
CXCL13	0.721 (0.615–0.828)	7
WISP-1	0.720 (0.625–0.814)	8
FR-alpha	0.719 (0.632–0.805)	9
MMP-1	0.710 (0.610–0.810)	10
MK	0.709 (0.608–0.810)	11
IL10	0.707 (0.613–0.801)	12
SCF	0.707 (0.605–0.809)	13
TNFRSF6B	0.706 (0.605–0.807)	14
CTSV	0.702 (0.597–0.807)	15

**Table 8:** Three-biomarker models. Benign tumors vs. borderline + cancer.

Reference model	AUC (95%)	Rank	p-value^a^	Sensitivity at 95% specificity	Specificity at 95% sensitivity
HE4+CA-125+age	0.770 (0.674–0.865)	-	-	0.172 (0.035–0.483)	0.196 (0.105–0.629)
Additional marker					
ITGAV	0.818 (0.737–0.900)	1	0.047	0.276 (0.103–0.517)	0.441 (0.168–0.650)
VEGFR-2	0.816 (0.725–0.908)	2	0.132	0.345 (0.103–0.586)	0.252 (0.091–0.664)
DNER	0.808 (0.720–0.896)	3	0.075	0.241 (0.034–0.552)	0.476 (0.028–0.615)
CTSV	0.803 (0.710–0.897)	4	0.206	0.379 (0.207–0.586)	0.301 (0.105–0.587)
SCF	0.794 (0.702–0.885)	5	0.204	0.207 (0.034–0.483)	0.245 (0.063–0.643)
CXCL13	0.792 (0.708–0.875)	6	0.240	0.310 (0.138–0.517)	0.448 (0.231–0.601)
MMP-1	0.785 (0.694–0.876)	7	0.198	0.241 (0.069–0.484)	0.245 (0.077–0.671)
FR-alpha	0.777 (0.690–0.864)	8	0.533	0.172 (0.034–0.517)	0.343 (0.245–0.587)
IL10	0.774 (0.681–0.867)	9	0.364	0.172 (0.034–0.448)	0.217 (0.084–0.622)
IL6	0.774 (0.675–0.874)	10	0.628	0.276 (0.034–0.552)	0.119 (0.035–0.608)
WISP-1	0.772 (0.679–0.866)	11	0.661	0.207 (0.034–0.449)	0.252 (0.084–0.643)
MK	0.772 (0.677–0.867)	12	0.609	0.207 (0.034–0.517)	0.203 (0.112–0.615)
TNFRSF6B	0.772 (0.676–0.867)	13	0.672	0.207 (0.034–0.517)	0.238 (0.056–0.601)

^a^ Comparing the reference model and the reference model with an added biomarker.

**Fig 4:**
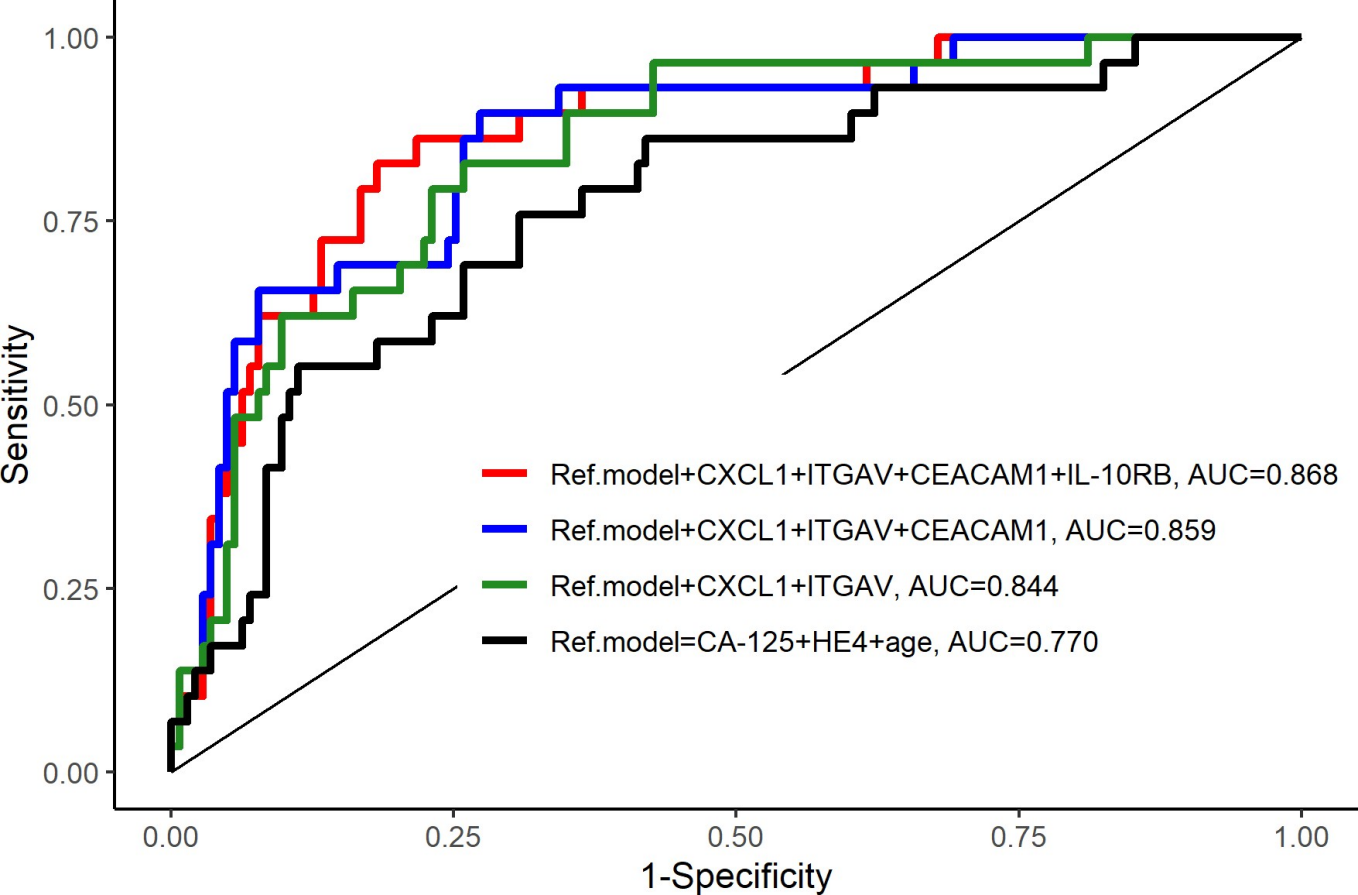
ROC curves for biomarker models. Benign tumors versus borderline + cancer.

Multi-marker models were developed using cross-validation and LASSO regression. A six-biomarker model (HE4, CA125, CXCL1, ITGAV, CEACAM1, IL-10RB and age) was the best model for discrimination between benign tumors and borderline tumors + cancer, with AUC 0.868 and sensitivity 0.86 / specificity 0.82 at best point cut-off (p = 0.016) (Table 9 and Fig 4).

**Table 9:** Biomarker models, LASSO selection. Benign tumors vs. borderline + cancer.

Reference model	AUC (95%)	p-value^a^	Sensitivity at 95% specificity	Specificity at 95% sensitivity	Specificity at best point	Sensitivity at best point se
HE4 + CA-125+ age	0.770 (0.674–0.865)	-	0.172 (0.035–0.483)	0.196 (0.105–0.615)	0.727 (0.532–0.930)	0.759 (0.517–0.966)
Additional marker combinations						
CXCL1+ITGAV+CEACAM1+IL-10RB	0.868 (0.799–0.937)	0.016	0.414 (0.103–0.724)	0.414 (0.266–0.818)	0.818 (0.664–0.930)	0.862 (0.690–0.966)
CXCL1+ITGAV+CEACAM1	0.859 (0.786–0.932)	0.015	0.482 (0.138–0.759)	0.371 (0.252–0.776)	0.755 (0.636–0.958)	0.897 (0.621–1.000)
CXCL1+ITGAV	0.844 (0.769–0.918)	0.017	0.355 (0.069–0.655)	0.559 (0.147–0.734)	0.748 (0.546–0.937)	0.862 (0.655–1.000)

^a ^Comparing reference model and model with added biomarkers.

## Discussion

### Our findings

In the current study only HE4 and MCA125 showed significant differences in expression levels in benign tumors vs. borderline + cancer, whereas six additional proteins differed significantly in expression levels between benign tumors and cancer. Also, the diagnostic performance of the reference model of HE4, CA125 and age was higher for benign tumors vs. cancer compared to benign tumors vs. borderline + cancer, illustrative of the diagnostic limitations of discriminating benign from borderline tumors using protein biomarkers. Biomarker levels may be normal or only slightly elevated in borderline tumors [11,27,28], and benign ovarian tumors as well as a range of benign conditions can present with elevated levels of biomarkers, including endometriosis, pelvic inflammatory disease, early pregnancy, and ascites of all causes [29,30]. While this is bound to lower the performance of biomarker-based algorithms, it can be argued whether this matters in a clinical setting, as borderline tumors have an excellent prognosis and will rarely progress to invasive cancer even after conservative surgery for a supposedly benign adnexal mass [31].

Integrin subunit Alpha V (ITGAV) was the only individual biomarker found to increase the performance of the reference model of HE4, CA125 and age above the significance threshold, for both comparisons. ITGAV is a subunit of the alpha V integrin receptor subfamily. Integrins are extracellular matrix proteins with a key role in angiogenesis. In epithelial ovarian cancer cells, ITGAV expression is essential for peritoneal dissemination [32]. Increased expression of ITGAV in tumor tissue has been associated with poor prognosis in ovarian cancer [33]. Interestingly, ITGAV expression was lower in plasma from patients with cancer compared with patients with benign tumors in our study, in line with the findings of Skubitz et al. who in their recent study on 92 biomarkers (Olink’s Oncology II panel) reported lower levels of ITGAV in serum from ovarian cancer patients compared to healthy women [34].

We were interested to see whether a combination of multiple biomarkers could further improve the diagnostic performance of our reference model of HE4, CA125 and age. In order to lower the risk for upwards bias, we used a combination of cross-validation and LASSO regression to select biomarkers for multivariate logistic regression models. The resulting models for discrimination between benign tumors and cancer and benign tumors vs. borderline + cancer differed considerably in their selection of biomarkers, further highlighting the diagnostic challenges of borderline tumors in ovarian cancer diagnostics. The best model to discriminate benign ovarian tumors from EOC including borderline tumors was the six-biomarker combination of HE4, CA125, ITGAV, CXCL1, CEACAM1, IL-10RB and age, performing with a diagnostic accuracy of AUC 0.868 and sensitivity 0.86 / specificity 0.82 at best point cut-off, compared to AUC 0.770 and sensitivity 0.76 / specificity 0.73 at best point for the reference model of HE4, CA125 and age.

CXCL1 is an inflammatory chemokine, promoting angiogenesis and recruitment of neutrophils [35,36]. Overexpression of CXCL1 induces EOC cell proliferation in vitro [37]. Wang et al 2011 reported CXCL1 to be overexpressed in serum from ovarian cancer patients and a biomarker model including CXCL1, CCL18 and CA125 was shown to discriminate ovarian cancer from benign ovarian tumors and healthy controls with a sensitivity of 92.6% for ovarian cancer together with impressively high specificity of 99% for healthy controls and 94% for benign tumors [38]. In line with the findings of Wang et al, CXCL1 was expressed in higher levels in plasma from ovarian cancer patients in our study.

CarcinoEmbryonic Antigen-related Cell Adhesion Molecule 1 (CEACAM1) is a member of the immunoglobulin superfamily of cell adhesion molecules (IgCAMs). CEACAM1 has important roles in angiogenesis, regulation of insulin action and immune responses and is crucial in the progression and metastasis of a range of cancers, exerting oncogenic as well as tumor suppressive actions [39,40]. Due to its inhibitory functions in immune cells including T and NK cells, in addition to being expressed on tumor cells, CEACAM1 makes a promising target for immunotherapy [41]. High expression of CEACAM1 correlates with better prognosis in advanced ovarian cancer patients, suggesting a tumor suppressor function in ovarian cancer [42]. In the current study plasma levels of CEACAM1 were lower in patients with cancer compared to benign tumors, supporting the role of CEACAM1 as a tumor suppressor.

Interleukin 10 receptor subunit B (IL-10RB / IL-10R2) is a subunit of the heterodimeric interleukin 10 receptor complex, expressed on most immune cells [43]. IL-10 is an important immunoregulatory cytokine [44,45]. The IL-10RB is also a subunit of the receptors for several other members of the IL-10-interferon family, including IL-22 [43]. IL-10RB is overexpressed in colorectal cancer and through binding of IL-22 contributes to colorectal carcinogenesis [46]. IL-10 levels are reported to be increased in serum and ascites from patients with ovarian cancer, with higher levels in advanced disease [47]. In the current study, the plasma levels of IL-10RB were higher in patients with cancer. To our knowledge, this is the first study reporting on circulating plasma levels of IL-10RB in patients with ovarian tumors.

In summary, biomarkers associated with both oncogenic (ITGAV, CEACAM1, CXCL1, IL-10RB) and tumor-suppressive actions (CEACAM1) were found to increase the diagnostic performance of HE4, CA125 and age in our study. Out of these markers, only ITGAV had AUC > 0.7 as an individual marker for discrimination between benign tumors and borderline tumors + cancer, indicating that biomarkers with poor performance individually can add valuable information in a multiple marker combination. Lower plasma levels were found for ITGAV and CEACAM1 in patients with cancer compared to patients with benign tumors. While the established biomarkers HE4 and CA125 are expressed at higher levels in EOC, the search for new diagnostic biomarkers should also include biomarkers expressed at lower levels in cancer.

### Biomarkers for ovarian cancer detection

In recent years, proximity extension assay (PEA) technology has been employed in the identification of novel protein biomarkers and biomarker combinations for early detection of ovarian cancer, with promising results [34,48–50]. The addition of inflammatory and immunological biomarkers to CA125 and HE4 holds potential to increase sensitivity and specificity compared to the established algorithms. However, in order for a screening method for a disease with incidence levels of ovarian cancer to be acceptable in a general population setting, a sensitivity of at least 75% and a specificity of 99.6%, corresponding to a PPV of 10% is recommended [51,52]. Adding to the difficulties in identifying a biomarker panel with high sensitivity and specificity for ovarian cancer is the heterogeneity of the disease, with different morphological subtypes expressing different patterns of biomarkers [53,54]. Also, as discussed above, borderline tumors and benign tumors can present with normal or slightly elevated biomarker levels [11,27–30].

Boylan et al. 2017 [48], in their study on 81 women (healthy controls, benign disease, early and late stage serous ovarian cancer), were able to increase sensitivity for the detection of early stage serous cancer versus healthy women from 0.93 (CA125 alone) to 0.95 (specificity 0.95) with a 12-protein classifier derived from analysis of the Olink’s Oncology Iv2 panel of 92 proteins (CA125, CD40.L, CD69, CXCL9, CXCL13, EGFR, EpCAM, DJ-1(PARK7), SELE, LAP.TGF.beta.1, TF, and VEGFR2) [48]. The same group recently published a study on 61 patients with late stage high-grade HGSC and 88 healthy controls analyzed with the Olink Oncology II panel. A multi-protein classifier of six biomarkers (CA125, FGFBP1, S100A4, EGF, ICOSLG, and MSLN) improved sensitivity from 0.85 (CA125 only) to 0.951 at a specificity of 0.996 to distinguish late stage HGSC from healthy women [34]. Enroth et al. in their recent large-scale study analyzing 593 plasma proteins with PEA, were able to identify a biomarker signature of 11 proteins (CA125, SPINT1, TACSTD2, CLEC6A, ICOSLG, MSMB, PROK1, CDH3, HE4, KRT19, and FR-alpha) plus age to discriminate ovarian cancer (all stages and histologies) from benign disease with a sensitivity of 0.85 at a specificity of 0.93 (AUC = 0.94, PPV = 0.92) [50].

The above referred PEA studies excluded borderline tumors, with the exception of Enroth et al. who did include borderline tumors in their final replication cohort and also included ovarian cancer samples of all histologies and stages [50]. The study by Boylan et al. 2017 [48] included serous cancer only, and Skubitz et al. 2019 [34] included late stage HGSC only [34,48]. The different study populations may explain the considerable variation in biomarkers included in the multi-protein models derived from the three studies. Only CA125 is included in all models. Excluding borderline tumors, early stage cancer and/or tumors of non-serous morphology is bound to strengthen the performance of a candidate biomarker panel. However, in the clinical setting these tumors will occur in the patient population with adnexal mass, which may also include (although rare) non-epithelial and metastatic tumors of the ovary. Given the vast heterogeneity of the tumor population it seems unlikely that a protein biomarker panel will reach a performance acceptable for screening on a population level.

In the common clinical challenge of risk assessment of an adnexal mass, HE4 and CA125 are validated for diagnostic use in the ROMA algorithm. A wide range of other protein biomarkers have been analyzed to date, with PEA and other diagnostic platforms. While a range of studies including ours indicate that some improvement in diagnostic accuracy can be gained from new biomarker combinations in comparison with CA125 and HE4, ultrasound-based models remain superior for assessing risk of malignancy in women with adnexal mass, albeit at the cost of lower specificity [14,18,55]. Biomarker tests can add improved specificity, are available at primary care level and complement diagnostic imaging in standard care today. In the triage of a patient with an adnexal mass, the patient with high risk of cancer according to a biomarker test can be referred to a tertiary center for further investigations including imaging with specialist ultrasound and/or CT/MR, before surgery. Still, histopathological examination of tissue is the gold standard for diagnosis.

### Strengths/Limitations

The heterogeneity of our small study population and the variations in sample size call for the statistical analyses to be interpreted with caution. Cross-validation was employed to reduce the risk of upwards bias from fitting and testing our multi-biomarker models on the same patient population, however, due to the single-cohort design we were not able to validate our models in a larger cohort. Further studies will be needed to validate our findings.

## Conclusion

HE4 was the best performing biomarker for discrimination of benign tumors versus EOC including borderline tumors in our study. ITGAV was the only individual biomarker found to improve the diagnostic performance of HE4, CA125 and age. Using LASSO regression, a multiplex model including 6 biomarkers (HE4, CA125, ITGAV, CXCL1, CEACAM1, IL-10RB) and age had the highest diagnostic accuracy for discrimination between benign ovarian tumors and EOC including borderline tumors. We find that the addition of other known carcinogenesis-related biomarkers in multiple marker combinations has potential to improve the performance of the established markers HE4 and CA125.

## Supporting information

S1 Fig Hierarchical cluster analysis. a) All patients b) Serous tumors only.
(TIF)

S2 Fig Principal component analysis. a) All patients b) Serous tumors only.
(TIF)

S3 Fig Box plots NPX values. Benign tumors, early and late stage EOC.
(TIFF)

S4 Fig Box plots NPX values. Benign tumors, borderline tumors, early and late stage EOC.
(TIF)

S1 Table Olink Oncology II panel. (DOCX)

S2 Table Olink Inflammation panel. (DOCX)

S1 File Olink Oncology II panel biomarkers. (DOCX)

S2 File Olink Inflammation panel biomarkers. (DOCX)
