# Supplementary figures and images for: A multiplex biomarker assay improves the diagnostic performance of HE4 and CA125 in ovarian tumor patients
Source: PLoS One. 2020 Oct 19;15(10):e0240418. doi: 10.1371/journal.pone.0240418 (PMC7571712; doi:10.1371/journal.pone.0240418)

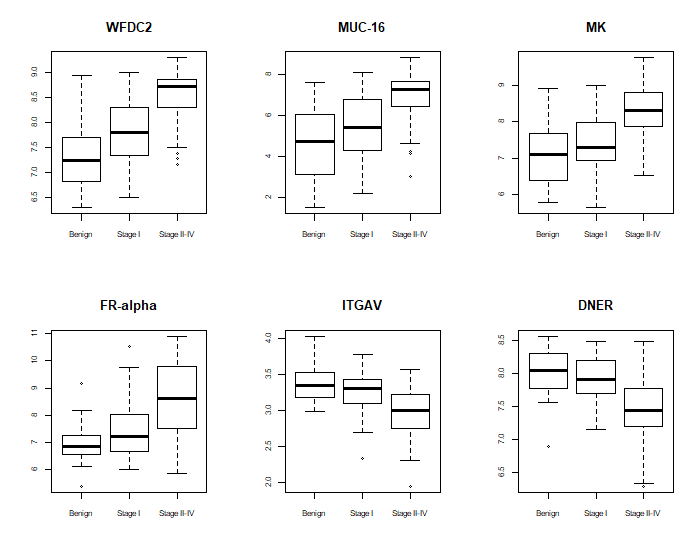

Supplement: S3 Fig — Benign tumors, early and late stage EOC. (TIFF) [file pone.0240418.s003.tiff]
