# Supplementary material for: A multiplex biomarker assay improves the diagnostic performance of HE4 and CA125 in ovarian tumor patients
Source: PLoS One. 2020 Oct 19;15(10):e0240418. doi: 10.1371/journal.pone.0240418 (PMC7571712; doi:10.1371/journal.pone.0240418)
Supplement: S1 Table — (DOCX) [file pone.0240418.s005.docx]

**S1 Table. Olink Oncology II panel.**

| **Assays** |  |
| --- | --- |
| **Name** | **Short Name** |
| Alpha-taxilin | TXLNA |
| Vascular endothelial growth factor A | VEGFA |
| Carboxypeptidase E | CPE |
| Kallikrein-13 | KLK13 |
| Carcinoembryonic antigen-related cell adhesion molecule 1 | CEACAM1 |
| Mesothelin | MSLN |
| Tumor necrosis factor ligand superfamily member 13 | TNFSF13 |
| Pro-epidermal growth factor | EGF |
| Tumor necrosis factor receptor superfamily member 6B | TNFRSF6B |
| Syndecan-1 | SYND1 |
| TGF-beta receptor type-2 | TGFR-2 |
| Interleukin-6 | IL6 |
| CD48 antigen | CD48 |
| Secretory carrier-associated membrane protein 3 | SCAMP3 |
| T-lymphocyte surface antigen Ly-9 | LY9 |
| Interferon gamma receptor 1 | IFN-gamma-R1 |
| Integrin alpha-V | ITGAV |
| TNF-related apoptosis-inducing ligand | TRAIL |
| Kallikrein-11 | hK11 |
| Glypican-1 | GPC1 |
| Tissue factor pathway inhibitor 2 | TFPI-2 |
| Kallikrein-8 | hK8 |
| Vascular endothelial growth factor receptor 2 | VEGFR-2 |
| Ly6/PLAUR domain-containing protein 3 | LYPD3 |
| Podocalyxin | PODXL |
| Protein S100-A4 | S100A4 |
| Insulin-like growth factor 1 receptor | IGF1R |
| Receptor tyrosine-protein kinase erbB-2 | ERBB2 |
| Receptor tyrosine-protein kinase erbB-3 | ERBB3 |
| Stem cell factor | SCF |
| SPARC | SPARC |
| Granzyme H | GZMH |
| Transforming growth factor alpha | TGF-alpha |
| Furin | FURIN |
| Protein CYR61 | CYR61 |
| Kallikrein-14 | hK14 |
| FAS-associated death domain protein | FADD |
| Methionine aminopeptidase 2 | MetAP 2 |
| Nectin-4 | PVRL4 |
| Tumor necrosis factor ligand superfamily member 6 | FASLG |
| Ephrin type-A receptor 2 | EPHA2 |
| Integrin beta-5 | ITGB5 |
| Galectin-1 | Gal-1 |
| Seizure 6-like protein | SEZ6L |
| Transmembrane glycoprotein NMB | GPNMB |
| Carbonic anhydrase 9 | CAIX |
| Melanoma-derived growth regulatory protein | MIA |
| Cathepsin L2 | CTSV |
| CD27 antigen | CD27 |
| Xaa-Pro aminopeptidase 2 | XPNPEP2 |
| Receptor tyrosine-protein kinase erbB-4 | ERBB4 |
| Hepatocyte growth factor | HGF |
| Disintegrin and metalloproteinase domain-containing protein 8 | ADAM 8 |
| 5'-nucleotidase | 5'-NT |
| Cyclin-dependent kinase inhibitor 1 | DKN1A |
| Delta-like protein 1 | DLL1 |
| Midkine | MK |
| Tyrosine-protein kinase ABL1 | ABL1 |
| Fibroblast growth factor-binding protein 1 | FGF-BP1 |
| Toll-like receptor 3 | TLR3 |
| Tyrosine-protein kinase Lyn | LYN |
| Proto-oncogene tyrosine-protein kinase receptor Ret | RET |
| Vimentin | VIM |
| Tumor necrosis factor receptor superfamily member 19 | TNFRSF19 |
| Cornulin | CRNN |
| T-cell leukemia / lymphoma protein 1A | TCL1A |
| CD160 antigen | CD160 |
| Tumor necrosis factor receptor superfamily member 4 | TNFRSF4 |
| MHC class I polypeptide-related sequence A and B | MIC-A/B |
| WNT1-inducible-signaling pathway protein 1 | WISP-1 |
| VEGF-co regulated chemokine 1 | CXL17 |
| Pancreatic prohormone | PPY |
| Protein S100-A11 | S100A11 |
| Amphiregulin | AREG |
| Endothelial cell-specific molecule 1 | ESM-1 |
| C-type lectin domain family 4 member K | CD207 |
| ICOS ligand | ICOSLG |
| WAP four-disulfide core domain protein 2 | WFDC2 |
| C-X-C motif chemokine 13 | CXCL13 |
| Mothers against decapentaplegic homolog 5 | MAD homolog 5 |
| A disintegrin and metalloproteinase with thrombospondin motifs 15 | ADAM-TS 15 |
| CD70 antigen | CD70 |
| R-spondin-3 | RSPO3 |
| Folate receptor gamma | FR-gamma |
| Carcinoembryonic antigen-related cell adhesion molecule 5 | CEACAM5 |
| Vascular endothelial growth factor receptor 3 | VEGFR-3 |
| Mucin-16 | MUC-16 |
| Wnt inhibitory factor 1 | WIF-1 |
| Granzyme B | GZMB |
| Fc receptor-like B | FCRLB |
| Annexin A1 | ANXA1 |
| Folate receptor alpha | FR-alpha |
|  |  |
