# Supplementary material for: A multiplex biomarker assay improves the diagnostic performance of HE4 and CA125 in ovarian tumor patients
Source: PLoS One. 2020 Oct 19;15(10):e0240418. doi: 10.1371/journal.pone.0240418 (PMC7571712; doi:10.1371/journal.pone.0240418)
Supplement: S2 Table — (DOCX) [file pone.0240418.s006.docx]

**S2 Table. Olink Inflammation panel.**

|  |  |
| --- | --- |
| **Assays** |  |
| **Name** | **Short Name** |
| Interleukin-8 | IL8 |
| Vascular endothelial growth factor A | VEGFA |
| Brain-derived neurotrophic factor | BDNF |
| Monocyte chemotactic protein 3 | MCP-3 |
| Glial cell line-derived neurotrophic factor | GDNF |
| CUB domain-containing protein 1 | CDCP1 |
| Natural killer cell receptor 2B4 | CD244 |
| Interleukin-7 | IL7 |
| Osteoprotegerin | OPG |
| Latency-associated peptide transforming growth factor beta 1 | LAP TGF-beta-1 |
| Urokinase-type plasminogen activator | uPA |
| Interleukin-6 | IL6 |
| Interleukin-17C | IL-17C |
| Monocyte chemotactic protein 1 | MCP-1 |
| Interleukin-17A | IL-17A |
| C-X-C motif chemokine 11 | CXCL11 |
| Axin-1 | AXIN1 |
| TNF-related apoptosis-inducing ligand | TRAIL |
| Interleukin-20 receptor subunit alpha | IL-20RA |
| C-X-C motif chemokine 9 | CXCL9 |
| Cystatin D | CST5 |
| Interleukin-2 receptor subunit beta | IL-2RB |
| Interleukin-1 alpha | IL-1 alpha |
| Oncostatin-M | OSM |
| Interleukin-2 | IL2 |
| C-X-C motif chemokine 1 | CXCL1 |
| Thymic stromal lymphopoietin | TSLP |
| C-C motif chemokine 4 | CCL4 |
| T cell surface glycoprotein CD6 isoform | CD6 |
| Stem cell factor | SCF |
| Interleukin-18 | IL18 |
| Signaling lymphocytic activation molecule | SLAMF1 |
| Transforming growth factor alpha | TGF-alpha |
| Monocyte chemotactic protein 4 | MCP-4 |
| Eotaxin-1 | CCL11 |
| Tumor necrosis factor ligand superfamily member 14 | TNFSF14 |
| Fibroblast growth factor 23 | FGF-23 |
| Interleukin-10 receptor subunit alpha | IL-10RA |
| Fibroblast growth factor 5 | FGF-5 |
| Matrix metalloproteinase-1 | MMP-1 |
| Leukemia inhibitory factor receptor | LIF-R |
| Fibroblast growth factor 21 | FGF-21 |
| C-C motif chemokine 19 | CCL19 |
| Interleukin-15 receptor subunit alpha | IL-15RA |
| Interleukin-10 receptor subunit beta | IL-10RB |
| Interleukin-22 receptor subunit alpha-1 | IL-22 RA1 |
| Interleukin-18 receptor 1 | IL-18R1 |
| Programmed cell death 1 ligand 1 | PD-L1 |
| Beta-nerve growth factor | Beta-NGF |
| C-X-C motif chemokine 5 | CXCL5 |
| TNF-related activation-induced cytokine | TRANCE |
| Hepatocyte growth factor | HGF |
| Interleukin-12 subunit beta | IL-12B |
| Interleukin-24 | IL-24 |
| Interleukin-13 | IL13 |
| Artemin | ARTN |
| Matrix metalloproteinase-10 | MMP-10 |
| Interleukin-10 | IL10 |
| Tumor necrosis factor | TNF |
| Interleukin-9 | IL9 |
| Vascular endothelial growth factor A | VEGFA |
| Brain-derived neurotrophic factor | BDNF |
| Monocyte chemotactic protein 4 | MCP-4 |
| Glial cell line-derived neurotrophic factor | GDNF |
| CUB domain-containing protein 2 | CDCP2 |
| Natural killer cell receptor 2B5 | CD245 |
| Interleukin-8 | IL8 |
| Osteoprotegerin | OPG |
| Latency-associated peptide transforming growth factor beta 2 | LAP TGF-beta-2 |
| Urokinase-type plasminogen activator | uPA |
| Interleukin-7 | IL7 |
| Interleukin-17C | IL-17C |
| Monocyte chemotactic protein 2 | MCP-2 |
| Interleukin-17A | IL-17A |
| C-X-C motif chemokine 12 | CXCL12 |
| Axin-1 | AXIN1 |
| TNF-related apoptosis-inducing ligand | TRAIL |
| Interleukin-20 receptor subunit alpha | IL-20RA |
| C-X-C motif chemokine 10 | CXCL10 |
| Cystatin D | CST6 |
| C-C motif chemokine 20 | CCL20 |
| Sulfotransferase 1A1 | ST1A1 |
| STAM-binding protein | STAMPB |
| Interleukin-5 | IL5 |
| Adenosine Deaminase | ADA |
| TNF-beta | TNFB |
| Macrophage colony-stimulating factor 1 | CSF-1 |
|  |  |
