# Supplementary material for: A multiplex biomarker assay improves the diagnostic performance of HE4 and CA125 in ovarian tumor patients
Source: PLoS One. 2020 Oct 19;15(10):e0240418. doi: 10.1371/journal.pone.0240418 (PMC7571712; doi:10.1371/journal.pone.0240418)
Supplement: S1 File — (DOCX) [file pone.0240418.s007.docx]

| **Olink ONCOLOGY II** |  |  |  |  |
| --- | --- | --- | --- | --- |
| **Short Name: ONCII** |  |  |  |  |
|  |  |  |  |  |
| **Assays** |  |  |  |  |
| **Name** | **Short Name** | **Uniprot ID** | **Olink ID** |  |
| Alpha-taxilin | TXLNA | P40222 | OID00655 |  |
| Vascular endothelial growth factor A | VEGFA | P15692 | OID00656 |  |
| Carboxypeptidase E | CPE | P16870 | OID00657 |  |
| Kallikrein-13 | KLK13 | Q9UKR3 | OID00658 |  |
| Carcinoembryonic antigen-related cell adhesion molecule 1 | CEACAM1 | P13688 | OID00659 |  |
| Mesothelin | MSLN | Q13421 | OID00660 |  |
| Tumor necrosis factor ligand superfamily member 13 | TNFSF13 | O75888 | OID00661 |  |
| Pro-epidermal growth factor | EGF | P01133 | OID00662 |  |
| Tumor necrosis factor receptor superfamily member 6B | TNFRSF6B | O95407 | OID00663 |  |
| Syndecan-1 | SYND1 | P18827 | OID00664 |  |
| TGF-beta receptor type-2 | TGFR-2 | P37173 | OID00665 |  |
| Interleukin-6 | IL6 | P05231 | OID00666 |  |
| CD48 antigen | CD48 | P09326 | OID00667 |  |
| Secretory carrier-associated membrane protein 3 | SCAMP3 | O14828 | OID00668 |  |
| T-lymphocyte surface antigen Ly-9 | LY9 | Q9HBG7 | OID00669 |  |
| Interferon gamma receptor 1 | IFN-gamma-R1 | P15260 | OID00670 |  |
| Integrin alpha-V | ITGAV | P06756 | OID00671 |  |
| TNF-related apoptosis-inducing ligand | TRAIL | P50591 | OID00672 |  |
| Kallikrein-11 | hK11 | Q9UBX7 | OID00673 |  |
| Glypican-1 | GPC1 | P35052 | OID00674 |  |
| Tissue factor pathway inhibitor 2 | TFPI-2 | P48307 | OID00675 |  |
| Kallikrein-8 | hK8 | O60259 | OID00676 |  |
| Vascular endothelial growth factor receptor 2 | VEGFR-2 | P35968 | OID00677 |  |
| Ly6/PLAUR domain-containing protein 3 | LYPD3 | O95274 | OID00678 |  |
| Podocalyxin | PODXL | O00592 | OID00679 |  |
| Protein S100-A4 | S100A4 | P26447 | OID00680 |  |
| Insulin-like growth factor 1 receptor | IGF1R | P08069 | OID00681 |  |
| Receptor tyrosine-protein kinase erbB-2 | ERBB2 | P04626 | OID00682 |  |
| Receptor tyrosine-protein kinase erbB-3 | ERBB3 | P21860 | OID00683 |  |
| Stem cell factor | SCF | P21583 | OID00684 |  |
| SPARC | SPARC | P09486 | OID00685 |  |
| Granzyme H | GZMH | P20718 | OID00686 |  |
| Transforming growth factor alpha | TGF-alpha | P01135 | OID00687 |  |
| Furin | FURIN | P09958 | OID00688 |  |
| Protein CYR61 | CYR61 | O00622 | OID00689 |  |
| Kallikrein-14 | hK14 | Q9P0G3 | OID00690 |  |
| FAS-associated death domain protein | FADD | Q13158 | OID00691 |  |
| Methionine aminopeptidase 2 | MetAP 2 | P50579 | OID00692 |  |
| Nectin-4 | PVRL4 | Q96NY8 | OID00693 |  |
| Tumor necrosis factor ligand superfamily member 6 | FASLG | P48023 | OID00694 |  |
| Ephrin type-A receptor 2 | EPHA2 | P29317 | OID00695 |  |
| Integrin beta-5 | ITGB5 | P18084 | OID00696 |  |
| Galectin-1 | Gal-1 | P09382 | OID00697 |  |
| Seizure 6-like protein | SEZ6L | Q9BYH1 | OID00698 |  |
| Transmembrane glycoprotein NMB | GPNMB | Q14956 | OID00749 |  |
| Carbonic anhydrase 9 | CAIX | Q16790 | OID00700 |  |
| Melanoma-derived growth regulatory protein | MIA | Q16674 | OID00701 |  |
| Cathepsin L2 | CTSV | O60911 | OID00702 |  |
| CD27 antigen | CD27 | P26842 | OID00703 |  |
| Xaa-Pro aminopeptidase 2 | XPNPEP2 | O43895 | OID00704 |  |
| Receptor tyrosine-protein kinase erbB-4 | ERBB4 | Q15303 | OID00705 |  |
| Hepatocyte growth factor | HGF | P14210 | OID00706 |  |
| Disintegrin and metalloproteinase domain-containing protein 8 | ADAM 8 | P78325 | OID00707 |  |
| 5'-nucleotidase | 5'-NT | P21589 | OID00708 |  |
| Cyclin-dependent kinase inhibitor 1 | DKN1A | P38936 | OID00709 |  |
| Delta-like protein 1 | DLL1 | O00548 | OID00710 |  |
| Midkine | MK | P21741 | OID00711 |  |
| Tyrosine-protein kinase ABL1 | ABL1 | P00519 | OID00712 |  |
| Fibroblast growth factor-binding protein 1 | FGF-BP1 | Q14512 | OID00713 |  |
| Toll-like receptor 3 | TLR3 | O15455 | OID00714 |  |
| Tyrosine-protein kinase Lyn | LYN | P07948 | OID00715 |  |
| Proto-oncogene tyrosine-protein kinase receptor Ret | RET | P07949 | OID00716 |  |
| Vimentin | VIM | P08670 | OID00717 |  |
| Tumor necrosis factor receptor superfamily member 19 | TNFRSF19 | Q9NS68 | OID00718 |  |
| Cornulin | CRNN | Q9UBG3 | OID00719 |  |
| T-cell leukemia / lymphoma protein 1A | TCL1A | P56279 | OID00720 |  |
| CD160 antigen | CD160 | O95971 | OID00721 |  |
| Tumor necrosis factor receptor superfamily member 4 | TNFRSF4 | P43489 | OID00722 |  |
| MHC class I polypeptide-related sequence A and B | MIC-A/B | Q29983,Q29980 | OID00723 |  |
| WNT1-inducible-signaling pathway protein 1 | WISP-1 | O95388 | OID00724 |  |
| VEGF-co regulated chemokine 1 | CXL17 | Q6UXB2 | OID00725 |  |
| Pancreatic prohormone | PPY | P01298 | OID00726 |  |
| Protein S100-A11 | S100A11 | P31949 | OID00727 |  |
| Amphiregulin | AREG | P15514 | OID00728 |  |
| Endothelial cell-specific molecule 1 | ESM-1 | Q9NQ30 | OID00729 |  |
| C-type lectin domain family 4 member K | CD207 | Q9UJ71 | OID00730 |  |
| ICOS ligand | ICOSLG | O75144 | OID00731 |  |
| WAP four-disulfide core domain protein 2 | WFDC2 | Q14508 | OID00732 |  |
| C-X-C motif chemokine 13 | CXCL13 | O43927 | OID00733 |  |
| Mothers against decapentaplegic homolog 5 | MAD homolog 5 | Q99717 | OID00734 |  |
| A disintegrin and metalloproteinase with thrombospondin motifs 15 | ADAM-TS 15 | Q8TE58 | OID00735 |  |
| CD70 antigen | CD70 | P32970 | OID00736 |  |
| R-spondin-3 | RSPO3 | Q9BXY4 | OID00737 |  |
| Folate receptor gamma | FR-gamma | P41439 | OID00738 |  |
| Carcinoembryonic antigen-related cell adhesion molecule 5 | CEACAM5 | P06731 | OID00739 |  |
| Vascular endothelial growth factor receptor 3 | VEGFR-3 | P35916 | OID00740 |  |
| Mucin-16 | MUC-16 | Q8WXI7 | OID00741 |  |
| Wnt inhibitory factor 1 | WIF-1 | Q9Y5W5 | OID00742 |  |
| Granzyme B | GZMB | P10144 | OID00743 |  |
| Fc receptor-like B | FCRLB | Q6BAA4 | OID00744 |  |
| Annexin A1 | ANXA1 | P04083 | OID00745 |  |
| Folate receptor alpha | FR-alpha | P15328 | OID00746 |  |
|  |  |  |  |  |
|  |  |  |  |  |
|  |  |  |  |  |
|  |  |  |  |  |
|  |  |  |  |  |
|  |  |  |  |  |
|  |  |  |  |  |
