# Supplementary material for: A multiplex biomarker assay improves the diagnostic performance of HE4 and CA125 in ovarian tumor patients
Source: PLoS One. 2020 Oct 19;15(10):e0240418. doi: 10.1371/journal.pone.0240418 (PMC7571712; doi:10.1371/journal.pone.0240418)
Supplement: S2 File — (DOCX) [file pone.0240418.s008.docx]

| **Olink INFLAMMATION** |  |  |  |  |
| --- | --- | --- | --- | --- |
| **Short Name: INF** |  |  |  |  |
|  |  |  |  |  |
| **Assays** |  |  |  |  |
| **Name** | **Short Name** | **Uniprot ID** | **Olink ID** |  |
| Interleukin-8 | IL8 | P10145 | OID00471 |  |
| Vascular endothelial growth factor A | VEGFA | P15692 | OID00472 |  |
| Brain-derived neurotrophic factor | BDNF | P23560 | OID00473 |  |
| Monocyte chemotactic protein 3 | MCP-3 | P80098 | OID00474 |  |
| Glial cell line-derived neurotrophic factor | GDNF | P39905 | OID00475 |  |
| CUB domain-containing protein 1 | CDCP1 | Q9H5V8 | OID00476 |  |
| Natural killer cell receptor 2B4 | CD244 | Q9BZW8 | OID00477 |  |
| Interleukin-7 | IL7 | P13232 | OID00478 |  |
| Osteoprotegerin | OPG | O00300 | OID00479 |  |
| Latency-associated peptide transforming growth factor beta 1 | LAP TGF-beta-1 | P01137 | OID00480 |  |
| Urokinase-type plasminogen activator | uPA | P00749 | OID00481 |  |
| Interleukin-6 | IL6 | P05231 | OID00482 |  |
| Interleukin-17C | IL-17C | Q9P0M4 | OID00483 |  |
| Monocyte chemotactic protein 1 | MCP-1 | P13500 | OID00484 |  |
| Interleukin-17A | IL-17A | Q16552 | OID00485 |  |
| C-X-C motif chemokine 11 | CXCL11 | O14625 | OID00486 |  |
| Axin-1 | AXIN1 | O15169 | OID00487 |  |
| TNF-related apoptosis-inducing ligand | TRAIL | P50591 | OID00488 |  |
| Interleukin-20 receptor subunit alpha | IL-20RA | Q9UHF4 | OID00489 |  |
| C-X-C motif chemokine 9 | CXCL9 | Q07325 | OID00490 |  |
| Cystatin D | CST5 | P28325 | OID00491 |  |
| Interleukin-2 receptor subunit beta | IL-2RB | P14784 | OID00492 |  |
| Interleukin-1 alpha | IL-1 alpha | P01583 | OID00493 |  |
| Oncostatin-M | OSM | P13725 | OID00494 |  |
| Interleukin-2 | IL2 | P60568 | OID00495 |  |
| C-X-C motif chemokine 1 | CXCL1 | P09341 | OID00496 |  |
| Thymic stromal lymphopoietin | TSLP | Q969D9 | OID00497 |  |
| C-C motif chemokine 4 | CCL4 | P13236 | OID00498 |  |
| T cell surface glycoprotein CD6 isoform | CD6 | Q8WWJ7 | OID00499 |  |
| Stem cell factor | SCF | P21583 | OID00500 |  |
| Interleukin-18 | IL18 | Q14116 | OID00501 |  |
| Signaling lymphocytic activation molecule | SLAMF1 | Q13291 | OID00502 |  |
| Transforming growth factor alpha | TGF-alpha | P01135 | OID00503 |  |
| Monocyte chemotactic protein 4 | MCP-4 | Q99616 | OID00504 |  |
| Eotaxin-1 | CCL11 | P51671 | OID00505 |  |
| Tumor necrosis factor ligand superfamily member 14 | TNFSF14 | O43557 | OID00506 |  |
| Fibroblast growth factor 23 | FGF-23 | Q9GZV9 | OID00507 |  |
| Interleukin-10 receptor subunit alpha | IL-10RA | Q13651 | OID00508 |  |
| Fibroblast growth factor 5 | FGF-5 | Q8NF90 | OID00509 |  |
| Matrix metalloproteinase-1 | MMP-1 | P03956 | OID00510 |  |
| Leukemia inhibitory factor receptor | LIF-R | P42702 | OID00511 |  |
| Fibroblast growth factor 21 | FGF-21 | Q9NSA1 | OID00512 |  |
| C-C motif chemokine 19 | CCL19 | Q99731 | OID00513 |  |
| Interleukin-15 receptor subunit alpha | IL-15RA | Q13261 | OID00514 |  |
| Interleukin-10 receptor subunit beta | IL-10RB | Q08334 | OID00515 |  |
| Interleukin-22 receptor subunit alpha-1 | IL-22 RA1 | Q8N6P7 | OID00516 |  |
| Interleukin-18 receptor 1 | IL-18R1 | Q13478 | OID00517 |  |
| Programmed cell death 1 ligand 1 | PD-L1 | Q9NZQ7 | OID00518 |  |
| Beta-nerve growth factor | Beta-NGF | P01138 | OID00519 |  |
| C-X-C motif chemokine 5 | CXCL5 | P42830 | OID00520 |  |
| TNF-related activation-induced cytokine | TRANCE | O14788 | OID00521 |  |
| Hepatocyte growth factor | HGF | P14210 | OID00522 |  |
| Interleukin-12 subunit beta | IL-12B | P29460 | OID00523 |  |
| Interleukin-24 | IL-24 | Q13007 | OID00524 |  |
| Interleukin-13 | IL13 | P35225 | OID00525 |  |
| Artemin | ARTN | Q5T4W7 | OID00526 |  |
| Matrix metalloproteinase-10 | MMP-10 | P09238 | OID00527 |  |
| Interleukin-10 | IL10 | P22301 | OID00528 |  |
| Tumor necrosis factor | TNF | P01375 | OID00529 |  |
| C-C motif chemokine 23 | CCL23 | P55773 | OID00530 |  |
| T-cell surface glycoprotein CD5 | CD5 | P06127 | OID00531 |  |
| Macrophage inflammatory protein 1-alpha | CCL3 | P10147 | OID00532 |  |
| Fms-related tyrosine kinase 3 ligand | Flt3L | P49771 | OID00533 |  |
| C-X-C motif chemokine 6 | CXCL6 | P80162 | OID00534 |  |
| C-X-C motif chemokine 10 | CXCL10 | P02778 | OID00535 |  |
| Eukaryotic translation initiation factor 4E-binding protein 1 | 4E-BP1 | Q13541 | OID00536 |  |
| Interleukin-20 | IL-20 | Q9NYY1 | OID00537 |  |
| SIR2-like protein 2 | SIRT2 | Q8IXJ6 | OID00538 |  |
| C-C motif chemokine 28 | CCL28 | Q9NRJ3 | OID00539 |  |
| Delta and Notch-like epidermal growth factor-related receptor | DNER | Q8NFT8 | OID01213 |  |
| Protein S100-A12 | EN-RAGE | P80511 | OID00541 |  |
| CD40L receptor | CD40 | P25942 | OID00542 |  |
| Interleukin-33 | IL33 | O95760 | OID00543 |  |
| Interferon gamma | IFN-gamma | P01579 | OID00544 |  |
| Fibroblast growth factor 19 | FGF-19 | O95750 | OID00545 |  |
| Interleukin-4 | IL4 | P05112 | OID00546 |  |
| Leukemia inhibitory factor | LIF | P15018 | OID00547 |  |
| Neurturin | NRTN | Q99748 | OID00548 |  |
| Monocyte chemotactic protein 2 | MCP-2 | P80075 | OID00549 |  |
| Caspase 8 | CASP-8 | Q14790 | OID00550 |  |
| C-C motif chemokine 25 | CCL25 | O15444 | OID00551 |  |
| Fractalkine | CX3CL1 | P78423 | OID00552 |  |
| Tumor necrosis factor receptor superfamily member 9 | TNFRSF9 | Q07011 | OID00553 |  |
| Neurotrophin-3 | NT-3 | P20783 | OID00554 |  |
| Tumor necrosis factor (Ligand) superfamily, member 12 | TWEAK | O43508 | OID00555 |  |
| C-C motif chemokine 20 | CCL20 | P78556 | OID00556 |  |
| Sulfotransferase 1A1 | ST1A1 | P50225 | OID00557 |  |
| STAM-binding protein | STAMPB | O95630 | OID00558 |  |
| Interleukin-5 | IL5 | P05113 | OID00559 |  |
| Adenosine Deaminase | ADA | P00813 | OID00560 |  |
| TNF-beta | TNFB | P01374 | OID00561 |  |
| Macrophage colony-stimulating factor 1 | CSF-1 | P09603 | OID00562 |  |
|  |  |  |  |  |
|  |  |  |  |  |
|  |  |  |  |  |
|  |  |  |  |  |
|  |  |  |  |  |
|  |  |  |  |  |
|  |  |  |  |  |
